# Role of MSC in the Tumor Microenvironment

**DOI:** 10.3390/cancers12082107

**Published:** 2020-07-29

**Authors:** Ralf Hass

**Affiliations:** Biochemistry and Tumor Biology Laboratory, Department of Obstetrics and Gynecology, Hannover Medical School, 30625 Hannover, Germany; hass.ralf@mh-hannover.de; Tel.: +49-511-532-6070

**Keywords:** mesenchymal stroma-/stem-like cells, extracellular matrix, tumor microenvironment, cell interaction, cell fusion

## Abstract

The tumor microenvironment represents a dynamically composed matrix in which tissue-associated cancer cells are embedded together with a variety of further cell types to form a more or less separate organ-like structure. Constantly mutual interactions between cells of the tumor microenvironment promote continuous restructuring and growth in the tumor. A distinct organization of the tumor stroma also facilitates the formation of transient cancer stem cell niches, thereby contributing to progressive and dynamic tumor development. An important but heterogeneous mixture of cells that communicates among the cancer cells and the different tumor-associated cell types is represented by mesenchymal stroma-/stem-like cells (MSC). Following recruitment to tumor sites, MSC can change their functionalities, adapt to the tumor’s metabolism, undergo differentiation and synergize with cancer cells. Vice versa, cancer cells can alter therapeutic sensitivities and change metastatic behavior depending on the type and intensity of this MSC crosstalk. Thus, close cellular interactions between MSC and cancer cells can eventually promote cell fusion by forming new cancer hybrid cells. Consequently, newly acquired cancer cell functions or new hybrid cancer populations enlarge the plasticity of the tumor and counteract successful interventional strategies. The present review article highlights some important features of MSC within the tumor stroma.

## 1. Introduction

Human mesenchymal stroma-/stem-like cells (MSC) represent heterogeneous populations which can be derived e.g., from the tunica adventitia in perivascular regions of various adult organs and tissues such as bone marrow, adipose tissue, peripheral blood or dental pulp, among various others [1,2,3,4]. According to further nomenclature for MSC-like multipotent mesenchymal stromal cells or medicinal signaling cells, several cellular functions are associated with these cells, some of which are also controversially discussed [5]. These include distinct repair activity for damaged tissues [6], involvement in regenerative processes [7], immune-modulatory potential [8], neovascularization [9], paracrine activities, antimicrobial functions [10], and tumor-inhibitory [11] and tumor-promoting properties [12,13,14]. As compared to adult tissues, superior in vitro growth potential and improved regenerative capacity are attributed to neonatal human MSC isolated from birth-associated tissues such as the placenta, umbilical cord and amniotic membrane [1,15,16,17]. MSC are defined to share some common basic properties such as in vitro plastic adherence; simultaneous expression of the surface markers CD73, CD90 and CD105l; and in vitro differentiation capacity, at least along osteogenic, adipogenic and chondrogenic lineages [1,16,18]. In addition to these representative MSC characteristics, some cell types exhibit additional properties that are not shared by the remaining cell types. For example, umbilical cord-derived MSC produce and release higher amounts of TGF-β (transforming growth factor-β) and lower levels of VEGF-α (vascular endothelial growth factor-α) and EGF (epidermal growth factor) than adipose tissue-derived MSC and amnion-originating MSC, suggesting altered immune-modulative and pro-angiogenic potential among tissue-specific MSC (sub)populations [19]. Moreover, CD146-positive cells with MSC-like features in the bone marrow were characterized as hematopoiesis-supporting Angiopoietin-1-expressing osteoprogenitors displaying in vivo self-renewal capacity consistent with stem cell-like properties [20]. Whereas only a small subset of cells displays stem cell-like properties, MSC are considered heterogeneous, consisting of various interdependent subpopulations. Moreover, different organs exhibit tissue-specific environments, which adds to the variable characteristics of originating MSC.

The cellular environment plays an important role in further MSC development and contributes to heterogeneity. Many differences can also be induced artificially in vitro, e.g., during the isolation procedure for MSC by the application of either aberrant enzymatic digestion or explant culture, besides subsequent MSC expansion in xeno-free media, culture on rigid/stiff or on soft surfaces, passaging, and in vitro differentiation [21]. Furthermore, particular changes in the microenvironment such as low/high pH, hyperoxia/hypoxia/anoxia, low/high ion gradients and long-term culture promote variable conditions to enable the growth advantage of distinct MSC subpopulations, which can result in either increased heterogeneity or clonogenic convergence [22]. Although the growth properties of MSC primary cultures can be maintained for a limited time in vitro [23], permanently proliferating MSC-like cells represent a cell source with reproducible properties [24]. Thus, some characteristics of MSC and environmental conditions change during in vitro culture and may substantially differ from the in vivo situation.

Alterations in the microenvironment are also observed during tumor growth, whereby MSC play an important role in developing tropism towards tumors. The tumor microenvironment (TME) of solid tumors represents an orchestration of extracellular matrix (ECM) together with various different cell types forming an organ-like entity. Accordingly, solid tumors can be regarded as a complex organ consisting of cancer cells in distinct states of development (differentiated, progenitor or cancer stem-like cells) in combination with a variety of differentially organized cell types, establishing a modular immune status, contributing to tumor angiogenesis and blood vessel formation, and building an extracellular matrix, which enables the associated cell populations to communicate within the TME and mutually acquire new functionalities.

The invasive growth and proliferation of cancer cells causes lesions and local tissue damage. These tissue injuries promote a pro-inflammatory environment, which attracts various different immune cells [25]. Some immune cell functions can adapt to the tumorigenic environment, such as the conversion of monocytes/macrophages to so-called tumor-associated macrophages (TAMs). Due to their regenerative potential, MSC are also recruited to cancer cell-induced lesions to promote tissue repair. Following the activation of MSC paracrine activities, a variety of chemokines, growth factors and metabolites are secreted within the TME. These bioactive molecules can modulate the immune cell response. Besides this indirect pathway, MSC can also directly interact with immune cells, cancer cells and further populations within the TME such as endothelial cells to support tumor vasculogenesis [26]. Thus, using indirect and direct pathways, MSC establish a communication network within the tumor stroma involving both non-tumorigenic adjacent cells and cancer cells.

In particular, the crosstalk of MSC with cancer cells significantly changes the properties of these two cell populations. These cellular interactions can prompt cancer cells to mediate a differentiation program/the clonal selection of certain MSC subpopulations whereby these stroma-/stem-like cells adapt to the tumorigenic microenvironment by the acquisition of specialized functions. Thus, previous work suggested MSC were a mixture of different mutually interdependent subpopulations, all displaying the minimal criteria of a similar basic phenotype [24]. As a consequence, some MSC change their cell fate and acquire a spindle-like morphology accompanied by conversion to carcinoma-associated fibroblasts (CAFs) [27,28]. Indeed, the attraction of MSC to tumor sites involves an aberrant development of adjacent cell types, including the maturation of CAFs or development of TAMs, which build a fibrovascular network as part of the tumor-specific ECM.

## 2. Tumor Stroma

The tumor stroma describes the tumor ECM together with interacting cells of the TME. The ECM displays an important structural scaffold, which is primarily composed of structural proteins including proteoglycans, which predominantly contain hyaluronic acid and proteoglycans with chondroitin sulfate and heparin sulfate. Moreover, fibrous proteins such as collagens, fibronectin, cadherins, elastin, tenascin C and laminins display architectural structures, with the formation of fibers of different lengths and thicknesses within the ECM. In addition, various globular proteins of the IgG superfamily and integrins support intercellular communication within the ECM. Furthermore, growth factors, chemokines, cytokines, antibodies and metabolites majorly released by MSC contribute to a trophic environment within the tumor stroma to mutually affect the interacting cells. In particular, the stromal cell-derived factor 1 receptor CXCR4 and the matrix metalloproteinase MMP-2 are involved in the multistep migration processes of MSC tropism to the TME [29]. Moreover, ECM proteases such as urokinase plasminogen activator and its soluble receptor are activated at injury sites of cancer cell invasion and contribute to the attraction of interacting MSC in coordination with interleukins (IL-6 and IL-8) and monocyte chemoattractant protein-1 (MCP-1 = CCL2) [30] (Table 1).

The complex ECM structures of the tumor tissue together with interacting cells of the TME may also build a barrier to chemotherapeutic interventions, mimicking certain drug resistance phenomena [31]. While these different ECM components vary according to remodeling activities within the tumor tissue, structural properties including the fiber network morphology, fiber thickness, amount of intrafibrillar cross-links and mesh size represent limiting factors for the migrating cancer cells. A predominant role of fiber building and subsequent crosslinking by lysyl oxidases is attributed to different collagens representing the most abundant fibrous proteins within the ECM. Lysyl oxidases represent copper-dependent amine oxidases that catalyze the crosslinking of collagens, elastin and fibrillin in the ECM to form a network of fibers and thereby increase matrix stiffening, which affects tumor growth [32]. TME-interacting MSC play an important role in this architecture and the activation of lysyl oxidases, e.g., bone marrow-derived human MSC promote lysyl oxidase production from human breast carcinoma cells, which contributes to enhanced metastasis [33] (Table 1). Lysyl oxidase enzymatic activity can generate hydrogen peroxide as a metabolic product. If hydrogen peroxide concentrations cannot be sufficiently metabolized within the ECM, local accumulation can stimulate the small GTPase Rac1, leading to the enhanced migratory and invasive activity of cancer cells [34]. Thus, the cross-linking of collagens and further matrix proteins or tight association with elastins, laminins or fibronectin has been associated with cancer invasion and metastasis [35]. This is accompanied by increasing rigidity and stiffness of the tumor stroma. In turn, this causes elevated intracellular contractions of cytoskeletal proteins and a more rigid trabecular system of actin/myosin components within the cancer cells, promoting higher migratory capacity.

At a more molecular level, he activation of the small GTPases Rac1 and Cdc42, which are key effectors of the actin cytoskeleton protrusion machinery, reorganizes the actin cytoskeleton and promotes an actin-mediated cell motility [36]. This is based on an extracellular ICAM-1–MUC1 interaction, which confers intracellular Src kinase activation. Subsequent Src-mediated phosphorylation of the cytoplasmic domain of MUC1 activates a direct recruitment of CrkL, binding via its SH2 domain. The SH2/SH3 adaptor protein CrkL is involved in the regulation of cell migration. This function is mediated through the association with guanine nucleotide exchange factors such as Dock180 to catalyze GTPase activation and GDP/GTP exchange by the stimulation of Rac1 and Cdc42 [36]. Accordingly, the enhanced motility of MUC1-carrying cancer cells supports a significantly elevated metastatic potential. Moreover, MSC interacting with cancer cells within the tumor stroma contribute to the alteration of matrix stiffness via the small GTPases Rho A and Cdc42 [37] (Table 1). Consequently, the biophysical architecture and structural composition of the ECM coupled with the biochemical properties of the tumor microenvironment influence the capability for and degree of cellular movement. These components also determine the migration strategy and efficiency of cancer cell invasion and metastatic potential [38]. The various mechanisms of cancer cells’ activities by interaction with MSC and other tumor-associated cells can alter the tumor stroma to make it a permissive and supportive environment—also termed a “reactive” tumor stroma [39].

In the course of ECM structuring and remodeling, tumor cells are capable of mechanically sensing an altered composition of the ECM, which is mediated by integrin-conferred signaling and downstream adhesion mechanosensor proteins such as p125^FAK^ focal adhesion kinase. Therefore, the reduction of soft tissue paralleled by an increasing rigidity and stiffness of the tumor stroma evokes focal adhesions, which elevates RhoA-mediated actomyosin contraction, and this tissue rigidity can further induce site-directed cell migration [40]. Thus, following the stimulation of cellular movement, cancer cells interact with components of the ECM and adjacent cell types, such as MSC, TAMs, CAFs, different immune cell subsets, adipocytes, cells of the vascular system (e.g., endothelial colony forming progenitors and endothelial cells), pericytes and MSC [38,41,42,43]. Of interest, MSC and CAFs are equipped with motor proteins and a proteolytic arsenal of matrix proteases. These functions enable them to interact with the ECM and respond to signals from the ECM by supporting the formation of unique structures such as muscle, bone, cartilage or other connective tissues [44].

During interaction with cancer cells, MSC can be induced to differentiate into CAFs or myofibroblasts. These maturation processes stabilize tumor tissue at primary and metastatic sites, contribute to chemoresistance and promote cancer stemness by the secretion of a specific set of paracrine factors [47]. In a prostate cancer model, certain stimuli such as TME-derived TGF-β1 can activate and covert tumor site-recruited MSC to develop a CAF-like phenotype [45]. Moreover, the adaptation of MSC to the ovarian TME was associated with functional changes including the enhanced expression of the bone morphogenic proteins BMP2, BMP4 and BMP6 [46] (Table 1). In a skin tumor model-mediated switch of MSC to CAFs, TME-derived TGF-β1 and the generation of reactive oxygen species induced phenotypic changes by the expression of alpha-smooth muscle actin. This was accompanied by the activation of protein kinase C, which can relay different pathways [48] and further downstream signaling cascades. As a result, the transdifferentiation of the CAFs to myofibroblasts was observed, with an increased production of growth factors and cytokines including hepatocyte growth factor (HGF), VEGF and IL-6 [49]. Moreover, CAFs can stimulate the progression of solid tumors by the secretion of IL-6 and SDF-1 (stromal cell-derived factor-1 = CXCL12). In addition, the elevated expression of the smooth muscle cell markers tenascin-C and alpha-smooth muscle actin by CAFs/myofibroblasts supports the restructuring of the extracellular matrix within the tumor microenvironment [39]. Previous work also demonstrated that MSC-derived CAFs promote tumor cell growth not only in vitro but also in a co-implantation model in vivo [27]. Further roles of CAFs in cooperation with TAMs following cancer cell interaction within the tumor stroma include changes within the ECM by either the increased production of matrix proteins or increased synthesis of ECM-digesting enzymes [50,51]. The induced changes in the tumor stroma and remodeling of ECM proteins confer increased matrix stiffness, whereby the cancer cells can lose polarity and become proliferative and invasive [35]. Other matrix-remodeling factors such as urokinase plasminogen activator and its inhibitor, also known as serpin E1, which are controlled by TGF-β1 expression, can relay proinvasive and prometastatic signals [52]. Thus, architectural changes in the altered ECM influence tumor progression. Moreover, these mechanisms can promote epithelial–mesenchymal-transition (EMT) by a switch from E-cadherin to N-cadherin expression, cancer cell dissemination, and subsequent metastasis [53]. Indeed, distinct MSC subpopulations contribute to metastasis by the down-modulation and/or cleavage of E-cadherin in cancer cells [54].

## 3. Tumor Organ and Vascularization

The emergence and evolvement of tumors before the building and expansion of organ-like structures are discussed and suggested by different hypotheses, including a hierarchical and a stochastic model or a retrodifferentiation program. These three different models try to provide explanations for neoplastic growth and the corresponding development of tumor-initiating cells.

Based on the hierarchical model, some progenitor and/or stem cells might evade a normal stem cell niche (SCN), escape from the normal regulation of proliferation, and establish apoptosis resistance and stemness by developing cancer progenitor cells (CPCs) or aberrant tumorigenic cancer stem-like cells (CSCs). While CPCs can generate different tumor subtypes, CSCs are characterized by their self-renewal capacity and contribute to tumor initiation and maintenance [55]. In addition to starting from a SCN, tumorigenesis can start from normal differentiated somatic cells after the stochastic or random acquisition of oncogenic mutations resulting in genomic instability with subsequent aberrant proliferation and hyperplastic expansion [56]. Alternatively, CSCs can develop by a retrodifferentiation process in normal progenitor cells or CPCs in which they acquire self-renewal capacity and maintain tumorigenicity by the establishment of a cancer stem cell niche (CSCN) [57,58,59]. Retrodifferentiation is characterized by distinct cell-specific signaling leading to a reversion of all differentiated properties back to a stem-like phenotype, including rejuvenation, which extends the multi-directional possibilities of cellular development [60,61].

The vascularization of tumor tissue represents an important property for the continuous nutrient support of the cancer cells. A fibroblast-like phenotype located within the tunica adventitia in perivascular regions of the diverse tissues presumably represents MSC. Moreover, pericytes that express various markers similar to MSC are also present in the vasculature.

The establishment of a tumor-associated extracellular matrix by cancer cells together with a variety of differentially organized cell types, including aberrant MSC such as CAFs and modified macrophages such as TAMs, creates a distinct immune status and contributes to blood vessel formation and neovascularization, which equips the associated cell populations for enhanced communication within the tumor stroma. These features represent the propensity of solid tumors to develop like a complex organ-like structure, similar to an invasive and uncontrolled growing organism within the body [45]. Besides CAFs, TAMs and further immune cells, pericytes, predominantly reside in perivascular niches of tumor blood vessels and can associate with endothelial colony-forming progenitors and vascular endothelial cells by promoting the elongation of tumor-associated blood capillaries. Although expressing a variety of markers similar to those of MSC, pericytes represent a more differentiated phenotype by enhanced gene expression patterns associated with smooth muscle cells and angiogenesis. However, in contrast to the normal healthy vasculature, tumor vessels display an abnormal physiology due to aberrant pericyte coverage and leaky endothelial cell layers. This inconsistent and discontinuous endothelium in tumor vessels fails to supply sufficient oxygen for the tumor stroma, causing increased hypoxia within the TME [62]. Accordingly, the metabolism of the cancer cells adapts to these special environmental conditions by alterations of energy metabolism. While normal cells primarily depend on energy storage and consumption via mitochondrial oxidative phosphorylation (OXPHOS), cancer cells focus on glycolysis, and the hypoxic conditions promote increased lactate production, as characterized by the Warburg effect. Likewise, MSC subtypes change functionality within a hypoxic environment by a more than 10-fold reduced oxygen consumption and increased lactate production, which is paralleled by enhanced cell growth and reduced apoptosis [63]. Within these tumor-specific environmental conditions, the MSC-mediated secretion of VEGF contributes to neovascularization [26,64]. The complex action of MSC on the tumor vasculature involves the paracrine activity of various secreted factors. Thus, the MSC-mediated release of proangiogenic factors such as VEGF and angiopoietin—in combination with IL-6, IL-8, TGF-β, PDGF, bFGF and FGF-7—directly stimulates the formation of blood vessels [3]. In breast and prostate cancer models, the presence of bone marrow-derived MSC correlated with a higher abundance of blood vessels by the promotion of tube formation [65]. Additionally, MSC and trophic factors secreted by MSC have been shown to enhance angiogenesis by the involvement of the activated Akt PI3-kinase pathway and downstream signaling via increased levels of phosphorylated Akt [66]. Moreover, MSC themselves can acquire an endothelial phenotype induced by TGF-β/JNK signaling and which is negatively regulated by p38α [67,68].

One of the predominant pro-tumoral functions of TAMs is also associated with their pro-angiogenic capabilities besides their requirement for tumor cell migration, invasion and metastasis [69]. TAMs generally accumulate in a hypoxic environment of the tumor stroma, and vice versa, hypoxia triggers a pro-angiogenic program in these cells. Accordingly, TAMs secrete a series of specific pro-angiogenic factors (VEGF, IL-1β, TNF-α, angiogenin and semaphorin 4D) promoting an angiogenic switch and neovascularization together with a more malignant transition of the tumor cells. Thus, previous work has demonstrated that the co-culture of macrophages together with cancer cells induces increased cell migration, which is promoted via the macrophage-mediated release of TNFα (tumor necrosis factor alpha) [70].

## 4. MSC and Immune Cell Function in the TME

Immune cells are present throughout the TME and include populations from the innate and adaptive immune system largely interacting with tumor tissue-associated MSC, whereby lymphocytes represent the majority of tumor-infiltrating immune cells [71]. These include CD4^+^ T_H_1 and T_H_2 cells, CD8^+^ cytotoxic T cells, regulatory T cells (T_regs_), various sub-populations of B cells, natural killer cells, dendritic cells, monocytes and macrophages, which can adapt to the tumorigenic environment by altering their activity status, e.g., by communicating with cancer cells [72]. The modulation of immune cell activity within the tumor stroma applies to the various T cell subsets, e.g., CD8^+^ T cells and CD4^+^ T_H_1 T cells predominantly exhibit anti-cancer effects whereby their strong infiltration and accumulation in various solid tumor tissues such as breast, ovarian, cervical, lung and colorectal cancers induces tumor reduction with a favorable patient prognosis. Conversely, CD4^+^ T_H_2 cells and T_regs_ suppress the immune response and contribute to the inhibition of anti-tumor activity.

MSC accumulate within the tumor stroma by changes in the environment such as elevated acidification, nutrient deprivation and increasing hypoxia. Furthermore, increasing cytokine concentrations of fibroblast growth factor-2, monocyte chemotactic protein-1 (= CCL2) and the pro-inflammatory IL-6 activate additional MSC recruitment [73]. The accumulation of other pro-inflammatory cytokines within the tumor microenvironment including TNFα contributes to additional MSC accumulation and promotes the enhanced migration and invasiveness of cancer cells in breast and ovarian tumors [74]. The interaction of immune cells with MSC within the tumor stroma can activate MSC’s immune-modulating capacity. MSC display unique immunologic characteristics and express very low levels of MHC (major histocompatibility complex) class I antigens and undetectable amounts of MHC class II antigens or costimulatory molecules such as CD40, CD80 and CD86, which protects them from alloreactive natural killer cell-mediated lysis [75]. On the other hand, MSC can secrete various immune-modulators, such as nitric oxide (NO), PGE2 (prostaglandin E2), IL-6, IL-10, metabolites of indoleamine 2,3-dioxygenase (IDO) and human leukocyte antigen-G, which is associated with the induction of tolerance and a shift from a T cell T_H_1 to T_H_2 immune response. In addition, MSC can inhibit effector T cell proliferation by activating apoptosis-like mechanisms via PD-1 (programmed cell death protein 1) and appropriate interaction with the corresponding ligands PD-L1 and PD-L2 [76]. Other T cell populations such as T_regs_ are affected by metabolites such as adenosine and PGE2 [77]. Besides the immune-suppressive and anti-inflammatory effects of MSC for regulatory T cells, MSC are also involved in converting macrophage activities, whereby further effects are attributed to metabolites of the tryptophan pathway [78]. The enzyme IDO—which is expressed, for example, by MSC—converts tryptophan by the generation of kynurenine. The release of the macrophage inflammatory proteins MIP-1α/CCL3 and MIP-2α/CXCL2 together with PGE2 and kynurenine by MSC contributes to the conversion of inflammatory M1 macrophages to alternatively activated, immunosuppressive M2 macrophages [79]. Whereas MSC can reprogram M1 macrophages to switch to M2 macrophages in response to these progressive stimuli, macrophages can also adapt to the environmental conditions of the altered physiological processes present in the tumor stroma. During this adaptation process within the tumor stroma, macrophages become TAMs, whereby these transformed tumor-associated cells can trigger further tumor development by supporting angiogenesis and ECM remodeling [80]. Together, these findings suggest that a timely available local concentration of certain metabolites confers the appropriate signals to trigger MSC for the modulation of various immune cell activities.

## 5. MSC and Cancer Cell Interactions—Cancer Stem Cell Niche

Besides communicating with different cell types within the tumor stroma, MSC can also indirectly and directly interact with cancer cells [14,43,81,82]. Based upon these tumor-homing properties, MSC are discussed as an ideal candidate for delivering anti-tumor agents, as a potential clinical approach [83].

**Figure 1:**
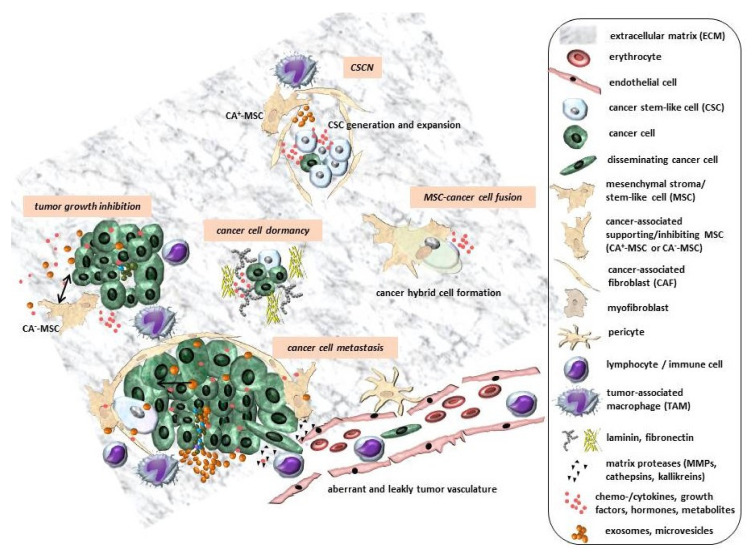
Different processes between MSC and cancer cells within the tumor stroma are mediated indirectly via the exchange of chemokines/cytokines, growth factors, metabolites such as PGE2 and exosomes/microvesicles. Certain restructuring of the ECM by proteases and the production of matrix proteins can form transient compartments to enable (1) metastasis by EMT and the trans-endothelial migration of disseminating cancer cells in tumor vessels displaying an abnormal physiology due to aberrant pericyte coverage and leaky endothelial cell layers; (2) cancer cell growth and inhibition by the contribution of CA^+^-MSC and CA^−^-MSC; (3) the formation of dynamic CSCNs for the generation, expansion and maintenance of CSCs; and (4) cancer hybrid cell formation by MSC–cancer cell entosis or cell fusion (adapted from [82]).

The release and exchange of various biological compounds including metabolites, proteins/peptides and circulating/cell-free DNA (cfDNA) act indirectly. CfDNA with very short (<200 bp) double-stranded DNA fragments can be derived not only from dead or necroptotic cells but also from cancer cells and cells of the TME to affect, for example, neighboring MSC by horizontal gene transfer [84]. Moreover, small extracellular vesicles such as microvesicles and exosomes can mediate indirect communication. In particular, the exchange of miRNA-carrying exosomes between MSC and cancer cells can mutually change cell functions (Figure 1). Cancer cell-derived exosomes contain various proteins and tumor-specific nucleic acids, which are transmitted and incorporated by nearby residing cells such as MSC. Consequently, MSC are re-programmed by a change in their normal trophic functionality to become pro-tumorigenic. Vice versa, the uptake of MSC-released exosomes by cancer cells can change the tumor functionality of several tumors including breast and ovarian cancers [13]. Thereby, MSC exosomes can confer signals that inhibit or promote tumor growth. Of interest, the mutual exchange of cargo by exosomes also provides useful non-cellular therapeutic vehicles for addressing primary and metastatic cancer cells. Indeed, previous work documented that taxol-loaded MSC-isolated exosomes significantly reduce lung and ovarian cancer cell growth and selectively target various organ metastases in breast carcinoma [85]. A clinical advantage of MSC-derived exosomes is that they also meet safety aspects, while the grafted cellular progenitors could raise some concerns in clinical setting.

The direct interaction of MSC with cancer cells can be mediated, among other ways, by:

- Conexin-based GJIC (gap junctional intercellular communication) [12];
- Notch receptor signaling involved in maintaining the self-renewal and amplification of CSCs [12,14];
- The formation of F-actin-rich nanotubes to exchange molecules and/or small organelles [14,82];
- Trogocytosis for the exchange of cell membrane patches with associated membrane proteins [14,82];
- Cell fusion, with the generation of new hybrid cancer cell populations [86,87].

However, the results of these MSC–cancer cell interactions are controversially discussed with respect to the tumor-inhibiting or tumor-promoting effects of MSC.

Tumor-inhibitory effects were observed after the subcutaneous application of MSC into melanomas. Simultaneously, the intercalation of MSC into microvessel walls and interaction with endothelial cells via GJIC was accompanied by the MSC-mediated production of reactive oxygen species, which promoted apoptosis and some capillary degeneration [88]. Besides aberrant pericyte coverage, this effect may also contribute to the abnormal physiology of the tumor vasculature. Moreover, MSC isolated from the dermal tissue of an aborted human fetus and immortalized with hTERT (human telomerase reverse transcriptase) were capable to inhibit hepatoma cell line-induced tumor growth in SCID mice, which involved the WNT/β-catenin signaling pathway [89]. In addition, the secretion of DKK-1 (dickkopf-1) by MSC, which acts as a negative regulator of the WNT/β-catenin pathway, was suggested to reduce tumor growth [90]. By contrast, tumor-promoting activities of MSC evolve following adaptation to the tumor environment and transformation into CAFs, which alters the tumor stroma and contributes to enhanced tumor growth [28]. In particular, signaling via the MSC/CAF-mediated release of SDF-1 and its binding to its corresponding receptor CXCR4 promotes the growth and angiogenesis of breast carcinomas [91]. Moreover, the CCL5 production of MSC during interaction with breast cancer cells and co-stimuli not only increased breast cancer growth but also promoted metastatic spreading [92].

Considering different tumor entities, MSC populations can enhance the initial tumor growth of ovarian cancer cells by inducing a filamentous tumor environment with an altered pattern of BMP expression accompanied by an increasing number of cancer stem cells [46]. Moreover, MSC can enhance small cell hypercalcemic ovary tumor growth and contribute to chemoresistance [31,93]. In addition, the enhanced growth and metastasis of pancreatic and colon carcinomas among various other tumor types are observed to be induced by MSC [94].

Although human MSC display no signs of spontaneous transformation in vitro, certain derailed MSC subpopulations may directly trigger neoplastic development. Several studies provide evidence that tumor types such as sarcomas originate from aberrant MSC. Sarcomas can be discriminated by bone tumors and soft tissue tumors. Ewing sarcoma represents a poorly differentiated tumor arising in bone but also in soft tissues. These sarcomas often display gene translocations and are suggested to originate from MSC-like populations [95]. Chondrosarcoma cells in different states of development share a lot of functional similarities with MSC maturation along the chondrogenic pathway. This leads to the suggestion that chondrosarcoma progression is paralleled by the deregulated chondrocyte differentiation of MSC [96]. Aberrant MSC of adipose tissue also play an important role in the sarcomagenesis of soft tissue tumors such as liposarcomas. These findings may link the type of aberrant MSC tissue origin to the sarcoma type. However, further studies suggested that benign MSC also reside in perivascular locations of sarcoma tumors [97]. In this context, the application of human MSC in an in vivo model of Kaposi’s sarcoma induced tumor-suppressive effects [98], which underscores the variable pro-tumorigenic and anti-tumorigenic effects of MSC.

Other work supported the MSC origin of bone tumors such as osteosarcoma with frequent aberrations in the genes encoding components of the P53 pathway. In particular, changes in the cell cycle regulatory cdkn2 gene were identified as the predominant mediators of a malignant transformation of MSC [99]. In addition, MSC stimulate osteosarcoma growth and induce metastases to lung tissue [100]. MSC can participate in metastasis formation, i.e., via the release of TGFβ. This growth factor is known to promote invasion and metastasis through the induction of EMT as a possible prerequisite. EMT represents a morphogenetic program during which the epithelial phenotype of the carcinoma cells is lost and replaced with a mesenchymal one [101]. Besides the downmodulation of E-cadherin, the secretion or MSC-mediated exogenous activation of ECM-restructuring matrix metalloproteinases and acquisition of mesenchymal markers such as N-cadherin, vimentin and fibronectin can accompany an EMT program. These regulatory events result in alterations in cell–cell and cell–matrix interactions, the loss of cell polarity, and the degradation of ECM molecules. Other components such as processing bodies containing ribonucleoprotein complexes can also contribute to cancer cell EMT [102]. Thus, the activation of cancer cell dissemination and subsequent formation of distal metastases via the MSC-stimulated expression of EMT markers can be triggered by Snail/Snai2 (Slug), Twist, vimentin and N-cadherin [103].

Together, these findings suggest that according to a variety of different tumor types, MSC display distinctly different roles. These effects are also associated with diversified regulatory functions of MSC in angiogenesis, immune modulation and apoptosis within the TME. MSC can act bi-directionally within the tumor stroma both as cancer-associated tumor-inhibitory MSC (CA^−^-MSC) and as tumor-supporting MSC (CA^+^-MSC). These opposite MSC functionalities may be expressed simultaneously in the same tumor tissues and strongly depend on the current status of the MSC and the type, threshold and synergy of local stimuli. Thereby, the availability of these distinct stimuli may be limited to small regional parts within the MSC’s vicinity. A resulting heterogeneous activation of MSC depending on their present localization in different compartments of the tumor tissue suggests that certain parts of the tumor are slowed in growth by CA^−^-MSC while other tumor parts are strongly proliferating as mediated by CA^+^-MSC. These diverse effects are consistent with the often-observed inhomogeneous tumor growth. In this context, previous work concluded that the overall net balance of these stimulatory activities determines the tumor-inhibitory or tumor-promoting effect of MSC [82].

Besides these opposite roles of MSC in heterogeneously functioning parts of the tumor stroma, MSC subpopulations also contribute to the establishment and maintenance of a CSCN to support CSC (re)generation and expansion [59]. CSCs can develop from primary tumors or metastases, from a retrodifferentiation process, or from cell fusion within a specialized microenvironment by a distinct cytokine/growth factor/metabolite composition in orchestration with the tumor stroma. Whereas tumor type-specific CSCs and appropriate subpopulations may exist, the predominant functions of CSC include self-renewal capacity for the maintenance of tumor growth, differentiation and developmental capacities, escape from immune surveillance and resistance to various chemotherapeutic agents and apoptosis. Hypotheses suggest that a distinct microenvironment for CSCs is required that favors the establishment and maintenance of a CSCN. MSC and CAFs together with the tumor vasculature and immune cells may primarily contribute to the structural components and certain compartmentalization of a CSCN within the tumor stroma (Figure 1). Within this structured CSCN, the accumulation of metabolites enhances a cascade of mutual cellular interactions and signaling pathways for the generation of CSCs [4]. In particular, cancer cell- and immune cell-derived cytokines including IL1 activate the cyclooxygenase-2/prostaglandin-E synthase-1 pathway in MSC for increased PGE2 production. This, in turn, acts in an autocrine fashion together with paracrine IL1′s effects for the further stimulation of MSC to secrete a panel of cytokines. The cytokine cocktail together with PGE2 accumulates on a local gradient and activates the Wnt/Frizzled/β-catenin signaling pathway in the cancer cells, which contributes to the development of CSCs [104]. Although the complexity of a CSCN is still poorly understood, other CSC-specific and CSC-dependent factors may be required; for example, IL8 signaling via the corresponding receptor CXCR1 appears to be crucial for the maintenance of a certain subtype of breast CSCs, since drug-mediated interference with the IL8/CXCR1 crosstalk and interruption of the associated intracellular signal transduction revealed a decrease in the breast CSC population by the induction of apoptosis/necroptosis [105]. The maintenance of breast CSCs within a CSCN also requires a quiescent status of CSCs and other more differentiated cancer cells. This transition to dormancy involves the accumulation of thrombospondin-1, which is detectable in wound matrices and in tumor stroma, where it functions as an inhibitor of angiogenesis. Conversely, the regained proliferative capacity of the different cancer cell populations is accompanied by neovasculature sprouting and the perivascular release of various growth factors [39,106]. In sum, the switch between the generation, expansion and dormancy of CSCs is based on dynamic structural and metabolic changes within a CSCN (Figure 1). These dynamic alterations can also disassemble CSCN structures at certain tumor parts, paralleled by re-organization at different locations within the tumor stroma, which supports tumor plasticity.

Close interactions for uniting MSC with cancer cells include entosis-like mechanisms [107] or cell fusion [108], which can result in either tumor reduction or promotion [87]. During cancer cell fusion with MSC, tumor plasticity can be further elevated (Figure 1). Successful cell fusion would require the two adjacent cell membranes of MSC and cancer cells to co-localize in close proximity by the extension of local lamellipodia-containing membrane protrusions. Accordingly, the reorganization of the actin cytoskeleton and activation of adhesion molecules enable the accumulation of transmembrane fusogenic proteins to facilitate subsequent cell fusion [109]. This is substantiated by the involvement of actin polymerization components such as formin for the formation of filopodia and Arp2/3 to generate branched filaments of lamellipodia, which provide a fusion-permissive environment for MSC with different breast cancer populations [110]. While initial signals may vary depending on the cell type, previous in vitro work suggested a crucial role of TNFα released by MSC and signal transfer via the TNFα receptor and NFkB in breast cancer cells in promoting cell fusion [111].

Although little is known about the precise molecular mechanisms that trigger cancer cell fusion as compared to physiological fusion processes such as the formation of multinucleated myocytes or syncytiotrophoblasts, a variety of indirect and direct studies substantiate the appearance of cancer hybrid cells in vivo [86,112,113,114,115]. Thus, the fusion of MSC with breast or ovarian cancer cells can generate different hybrids displaying an altered tumorigenicity and metastatic behavior. In addition, resistance to a variety of tumor therapeutic agents is modulated in certain MSC/cancer hybrid cell populations. Consequently, the fusion of different cancer cell types with MSC including breast [111,116,117] and ovarian cancer cells [93] can generate new neoplastic populations displaying altered tumorigenic properties and metastatic behavior, which contribute to progressively increasing tumor heterogeneity. Such increased tumor plasticity worsens therapeutic strategies and patient prognoses. Therefore, future clinical approaches require concepts that modulate the dialog between MSC, the TME and cancer cells by anti-tumor engineered MSC or their products.

## 6. Conclusions

The tumor stroma comprises a complex interactive system with coordinated signaling between cancer cells and interacting cells of the TME together with the ECM by invasive organ-like structures. In particular, the interaction of tumor-infiltrating MSC or macrophages with cancer cells followed by tumorigenic adaptation and corresponding conversion into CAFs and TAMs appears to be tumor-tissue-specific. Consequently, the functionalities of CAFs and TAMs are selective and differ among several types of tumors. Such differences in functionality even apply to molecules such as PGE2 in coordination with further signals among distinct tumor compartments. While PGE2 mediates the immune modulation of several T cell subsets and conversion of M1 into M2 macrophages, further crucial roles of PGE2 confer signaling via the Wnt/Frizzled/β-catenin pathway within the CSCN, contributing to the development of CSCs.

This complexity of microenvironment-dependent signaling predominantly involves MSC. The enhanced recruitment and accumulation of MSC in a tumorigenic microenvironment contributes to immune modulation, tumor angiogenesis and various modes of indirect and direct cancer cell interactions by a possible simultaneous inhibition (CA^−^-MSC) and promotion (CA^+^-MSC) of tumor growth at different sites and compartments of the tumor. A certain compartmentalization by MSC and CAFs supports reversible dynamic structures in a CSCN for the maintenance and propagation of CSCs. Other forms of MSC–cancer cell interaction such as cell fusion can also promote CSC development and select new cancer hybrid populations, which complicates therapeutic regimens and leads to unfavorable patient outcomes.

Certain disadvantages of the use of primary MSC in regenerative medicine such as the heterogeneity of (sub)populations, finite life expectancy, limited expansion potential and individual donor variability may be significantly reduced by standardizing MSC sources, e.g., by utilizing permanently proliferating MSC cell line models [24]. Nevertheless, MSC or MSC-derived exosomes, with their tropism towards tumors, are considered as potential delivery vehicles for novel cell-based anti-cancer agents. Consequently, the properties of CA^−^-MSC or engineered anti-tumor MSC as drug carriers may provide a successful platform of tools for appropriate evaluation in preclinical models and subsequent clinical trials.

## Figures and Tables

**Table 1:** Interaction of mesenchymal stroma-/stem-like cells (MSC) with the tumor microenvironment (TME).

MSC Source	Mediators	References
bone marrow, cord blood	CXCR4, MMP-2	[29]
bone marrow	lysyl oxidases	[33]
human fetal bones	urokinase plasminogen activator and its receptor, IL-6, IL-8, MCP-1	[30]
bone marrow	Rac-1, Rho A, Cdc42, p125FAK focal adhesion kinase	[37,40]
bone marrow	TGF-β1	[45]
human epithelial ovarian cancers	BMP2, BMP4, BMP6	[46]

## Abbreviations

CA^−^-MSC	tumor-inhibitory MSC
CA^+^-MSC	tumor-supporting MSC
CAFs	carcinoma-associated fibroblasts
CPCs	cancer progenitor cells
CSCs	cancer stem-like cells
(C)SCN	(cancer) stem cell niche
DKK-1	dickkopf-1
EGF	epidermal growth factor
ECM	extracellular matrix
EMT	epithelial–mesenchymal transition
GJIC	gap junctional intercellular communication
IDO	indole 2,3-dioxygenase
MHC	major histocompatibility complex
MSC	mesenchymal stroma-/stem-like cells
MMP	matrix metalloproteinase
PD-1	programmed cell death protein 1
PGE2	prostaglandin E2
TAMs	tumor-associated macrophages
TGF-β	transforming growth factor-β
TME	tumor microenvironment
TNFα	tumor necrosis factor alpha
T_regs_	regulatory T cells
VEGF-α	vascular endothelial growth factor-α
